# An *in planta*, *Agrobacterium*-mediated transient gene expression method for inducing gene silencing in rice (*Oryza sativa* L.) leaves

**DOI:** 10.1186/1939-8433-5-23

**Published:** 2012-08-31

**Authors:** Aurélie Andrieu, Jean Christophe Breitler, Christelle Siré, Donaldo Meynard, Pascal Gantet, Emmanuel Guiderdoni

**Affiliations:** CIRAD, UMR AGAP, TAA108/03, Av Agropolis, F-34398, Montpellier, Cedex 05 France; Université Montpellier II, UMR DIADE, F-34398, Montpellier, Cedex 05 France

**Keywords:** *Agrobacterium tumefaciens*, Gene silencing, Leaf agroinfection, *OsPDS*, *OsSRL1*, Rice, Transitivity, Cross silencing

## Abstract

**Background:**

Localized introduction and transient expression of T-DNA constructs mediated by agro-infiltration of leaf tissues has been largely used in dicot plants for analyzing the transitivity and the cell-to cell movement of the RNAi signal. In cereals, however, the morphology of the leaf and particularly the structure of the leaf epidermis, prevent infiltration of a bacterial suspension in cells by simple pressure, a method otherwise successful in dicots leaves. This study aimed at establishing a rapid method for the functional analysis of rice genes based on the triggering of RNA interference (RNAi) following *Agrobacterium*-mediated transient transformation of leaves.

**Results:**

Using an agro-infection protocol combining a wound treatment and a surfactant, we were able to obtain in a reliable manner transient expression of a T-DNA-borne uidA gene in leaf cells of japonica and indica rice cultivars. Using this protocol to transiently inhibit gene expression in leaf cells, we introduced hairpin RNA (hpRNA) T-DNA constructs containing gene specific tags of the phytoene desaturase (*OsPDS*) and of the SLENDER 1 (*OsSLR1*) genes previously proven to trigger RNAi of target genes in stable transformants. SiRNA accumulation was observed in the agro-infected leaf area for both constructs indicating successful triggering of the silencing signal. Accumulation of secondary siRNA was observed in both stably and transiently transformed leaf tissues expressing the HpRNA OsSLR1 construct. Gene silencing signalling was investigated in monitoring the parallel time course of OsPDS-derived mRNA and siRNA accumulation in the agro-infiltrated leaf area and adjacent systemic sectors. The sensitive RT-Q-PCR method evidenced a consistent, parallel decrease of OsPDS transcripts in both the agroinfiltred and adjacent tissues, with a time lag for the latter.

**Conclusions:**

These results indicate that the method is efficient at inducing gene silencing in the agro-infected leaf area. The transfer of low amounts of siRNA, probably occurring passively through the symplastic pathway from the agro-infected area, seemed sufficient to trigger degradation of target transcripts in the adjacent tissues. This method is therefore well suited to study the cell-to-cell movement of the silencing signal in a monocot plant and further test the functionality of natural and artificial miRNA expression constructs.

**Electronic supplementary material:**

The online version of this article (doi:10.1186/1939-8433-5-23) contains supplementary material, which is available to authorized users.

## Background

In dicots plants, agro-infection methods allow efficient transfer and transient expression of T-DNA vectors in leaf cells. Transient assays by agro-infiltration have thus been increasingly used as a simple and rapid method for assaying gene function (Wroblewski et al. 2005; Small 2007). Because they provide a rapid, versatile and convenient way for achieving a very high level of gene expression in a distinct and defined zone of the leaf, these *Agrobacterium*-mediated transient expression systems have been largely used for analyzing the induction of the RNA silencing process, thereby validating gene function through down regulation of gene expression - as illustrated recently in grapevine (Bertazzon et al. 2012)- and analyzing the mechanism of transitivity and movement of RNA silencing in leaves (e.g. Schöb et al. 1997, Voinnet 2001).

In rice, as in other cereals, the establishment of such a system is hindered by the monocot leaf structure which includes the presence of an epidermal cuticular wax and high silicium content preventing infiltration of bacterial suspension by simple pressure. Microprojectile bombardment has been used as an alternative delivery method to introduce expression construct in cereal leaf cells. As to gene silencing, bombardment-mediated introduction of dsRNA corresponding to endogenous genes or transgenes in cells of maize, barley and wheat leaves proved to induce interference with gene function but restricted to the single cell where the delivery is achieved (Schweizer et al. 2000). This stresses the need to establish a rapid, versatile and convenient protocol for achieving a very high level of gene expression *in planta* -e.g. in a distinct and defined zone of the cereal leaf.

Once established and among a range of applications, such a method could be used for triggering a localized, hpRNA-mediated silencing process and further study the transitivity and spreading of the silencing signal throughout the whole plant. RNA silencing is non-cell autonomous and operates through different pathways involving separate mechanisms and, probably, distinct signals (Voinnet 2005a; Brodersen and Voinnet 2006; Ghildiyal and Zamore 2009). The systemic silencing signal reflects with many evidences an antiviral defence mechanism in plants: non-cell autonomous silencing relies on a systemic signal that moves ahead or follows RNA viruses by the same way of propagation to initiate RNA silencing and prevent or delay viral infection (Voinnet 2005b). While RNA-silencing induction and RNA degradation have been studied in detail, much less is known about why and how RNA silencing moves from cell-to-cell and sometimes spreads systemically in plants. The answer to these questions seems to be closely related to the transitive RNA silencing observed in *C. elegans* and plants. In these organisms, RNA silencing can be amplified by a phenomenon called transitivity which increases the initial pool of siRNAs by producing secondary siRNAs corresponding to sequences located outside the primary targeted regions of a transcript (Nishikura 2001; Sijen et al., 2001). Himber et al. (2003) have proposed a model of RNA silencing cell-to-cell movement including a central role for transitivity. The vast majority of experimental data used to formulate this model of RNA silencing movement over cells and organism was accumulated in *N. benthamiana* and *A. thaliana*. Regarding transitivity of RNA silencing and signalling, experience on monocotyledonous plants remains fairly limited since the report of the conservation of the transitive RNA silencing machinery in rice by Miki et al. (2005).

The goal of this study was first to establish a standardized *Agrobacterium* transfection protocol for inducing transient gene expression in rice leaf cells. The second objective was to illustrate this method in inducing the silencing of two endogenous rice genes (*OsPDS* and *OsSRL1)* to investigate the transitivity and systemic spreading of the RNAi signal in a monocotyledonous leaf.

## Methods

### Plant Material and in planta agroinfection

Three varieties of rice (*Oryza sativa* L.) were used in this study: (i) the sub tropical *japonica* rice cv. Zhong Zuo321 from China, (ii) the temperate *japonica* rice cv. Nipponbare and (iii) the high value *indica* rice cv. IR64 from IRRI. Bacteria used for rice agroinfection were plated on solid AB medium (Chilton et al. 1974) containing 50 mg/l kanamycin sulfate and 75 mg/l rifampicin and incubated at 28°C for 3 days. The bacteria were then collected with a flat spatula and re-suspended in liquid agroinfection medium (R2 Basic (Sallaud et al. 2003) + 10 g/l glucose, 200 μM acetosyringone, 0,01% Silwet L-77, pH5.5) by vortexing to give an absorbance at 600 nm between 0.5 and 0.8. The antepenultimate emerged leaf of plantlets at tillering stage were mechanically wounded with multiple needles and immersed in the *Agrobacterium* suspension at 20°C for 30 to 60 min. The plantlets were grown for 2 to 3 days at 20°C and then placed under containment greenhouse growth conditions. *Agrobacterium*-mediated transformation of seed embryo-derived embryogenic callus was performed according to Sallaud et al. 2003.

### Gene specific tag (GST) and T-DNA constructs

**Figure 1:**
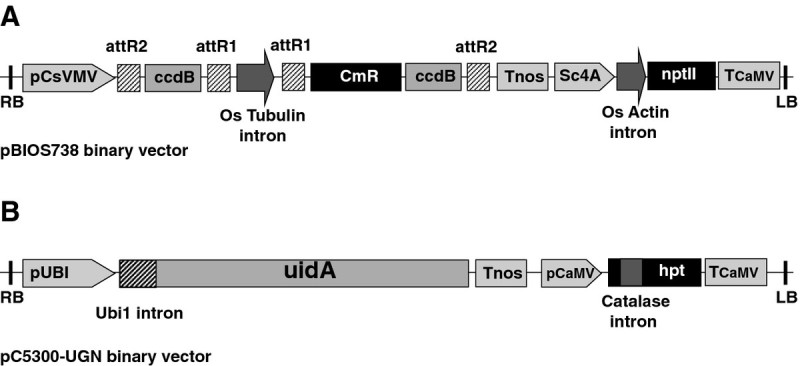
**Schematic maps of the pBIOS738 (W. Paul, Biogemma) and pC5300-UGN T-DNAs.** (**A**) The inverted repeat gateway cassette cloned between the *O. sativa* tubulin intron is under the control of the strong constitutive promoter of the cassava vein mosaic virus (CsVMV). Sc4A: promoter of the fourth subunit of the subterranean clover mosaic virus. nptII: neomycin phospho transferase II: Tnos : terminator of the *Agrobacterium* nopaline synthase gene: tCaMV : 3’ sequence of the Cauliflower Mosaic Virus (**B**) The core structure of the pC5300-UGN T-DNA. pUbi: promoter of the maize polyubiquitin 1 gene. *uidA*: *E. coli* β-glucuronidase coding sequence.

The binary vector pC5300, a pCAMBIA 1300 derivative, had been described elsewhere (Breitler et al. 2004). The reporter plasmid pC5300-UGN bears the *uidA* gene under the control of the entire 5’ untranslated region of the maize polyubiquitin gene *Ubi-1* (Christensen and Quail 1996) and the polyadenylation sequence from *A. tumefaciens nos* gene. The hpRNA binary vector pBIOS 738, kindly provided by W. Paul and P. Perez (BIOGEMMA, Clermont-Ferrand, France), was constructed by cloning the intron of rice tubulin1 gene between twin Gateway™ cassettes in inverted orientation into a pCAMBIA 2300 binary vector. This hpRNA expression cassette is under the control of the cassava vein mosaic virus (CsVMV) promoter (Verdaguer et al. 1998) and the polyadenylation sequence from *A. tumefaciens nos* gene (Figure 1).

For the rice *PDS* gene a 400 bp fragment (Accession no. AF049356.1, nucleotides no. 660–1040) was used as GST (forward primer: 5’-GAAGTTTGCTCTTGGACTTTTGCCA-3’ and reverse primer: 5’-TATTTGAGTTCCATCGGTAAGTGCA-3’).

For the *SLR1* gene (Accession no. AB030956), the GST (nucleotide 969–1469) is a DNA fragment of 500 bp (forward primer: 5’-AAGTTCGCCCACTTCACCGCAAATC-3’ and reverse primer: 5’-TCGAGGAATGAGCCGGAGTTGTGGT-3’). The hpRNA binary vectors were introduced into *Agrobacterium* strain EHA105 by electroporation.

### RNA analysis

Total RNA extraction was performed with Tri-Reagent (Sigma) and was used for both high-molecular-weight and small RNA analysis. Total RNA were separated on 17.5% denaturing polyacrylamid gels and then transferred to nylon membranes (Hybond NX, Amersham). Perfect-Hyb buffer (Sigma) was used for hybridization. Probes were DNA fragments labelled by random priming incorporation of [32 P]dCTP (Amersham). The GST probe was PCR products similar to GST cloned in the pBIOS738 binary vector. The 5’ probes were produced by PCR using the following set of primers: *5’pds*, 5’-GGTGCTTCGCAAGTAGCAGC-3’ and 5’-TACTAAGAAACAATGAAATG-3’ and 5’*slr1*, 5’-GTGCAAGGACAAGGTGATGG-3’ and 5’-GCACGCCCACTTCTACGAGT-3’. The 3’ probes were produced by PCR with the following set of primers used for RT-PCR analysis *3’-pds*, 5’-TTGTGCTCAGTCTGTAGTGGA-3’ and 5’-TGTGAAGGGATTAAGAGACC-3’ and *3’-slr1*, 5’-GGCACAATTGAAGCTTGACG-3’ and 5’-ATAGATGGGCTAGGAGGACCAAG-3’. Following hybridisation, the membrane was washed twice for 20 min at 50°C (2x SSC and 2% SDS) and all hybridisation signals were detected by phosphorimaging (Storm 820, Amersham).

### Real-time PCR analysis

Total RNA extraction was performed with Tri-Reagent (Sigma) according to the manufacturer’s instructions. The template DNA was removed by treatment with the DNA RQ1 RNase-free DNase kit (Promega). Each cDNA sample was subjected to real-time PCR analysis in triplicate. To normalize the variance among samples, *OsExp* (Os06g11070.1) was used as endogenous control (Caldana et al. 2007). Relative expression values were calculated after normalizing against the control cDNA. Primers were designed from 3' end of the gene using Primer3plus (http://www.bioinformatics.nl/cgi-bin/primer3plus/primer3plus.cgi) with QPCR parameters ‘on’. Each primer pair was checked for their specificity using the Primer Blaster tool in OryGenesDB (http://orygenesdb.cirad.fr/tools.html), which were further confirmed by dissociation curve analysis obtained after the QPCR reaction. First strand cDNA was synthesized by reverse transcription using 1.5 μg of total RNA in 20 μl of reaction volume using SuperScriptIII (Invitrogen) as per manufacturer's instructions. Diluted cDNA samples (1/10) were used for Real time PCR analysis with 200 mM of each gene specific primer mixed with SYBR Green PCR master mix in a final volume of 15 μl following manufacturer's instructions. The reaction was carried out in 96-well optical reaction plates (Roche), using the Light Cycler 480 Sequence Detection System and software (Roche).

Primer sequences were: *5’-pds*, 5’-GTTCCTGATCGAGTGAACGATG-3’, *3’-pds*, 5’-CGAACATGGTCAACAATAGGC-3’; *5’-OsExp*, 5’-CGGTTAGCTAGAGTTCATGTGAGA-3’, and *3’-OsExp*, 5’-ATTGGAGTAGTGGAGTGCCAAA-3’.

## Results

### Agroinfection of rice leaf tissues with an A. tumefaciens suspension

**Figure 2:**
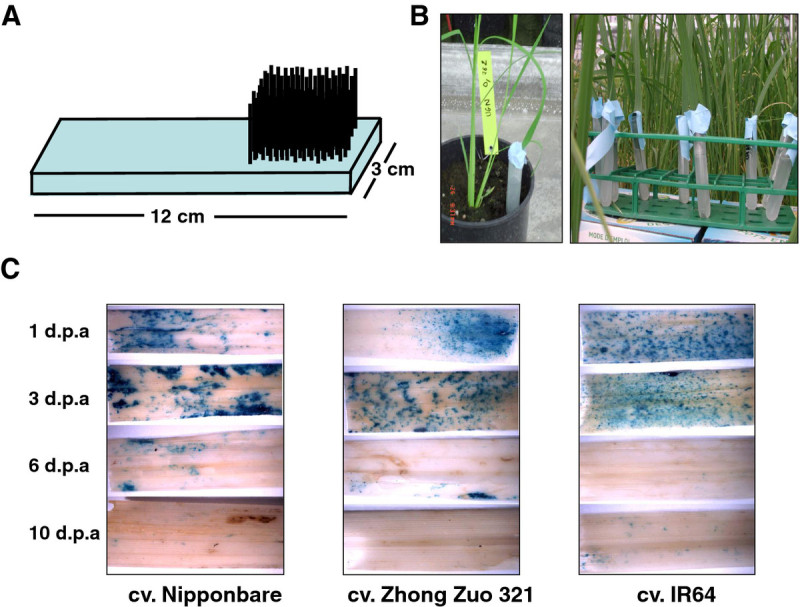
**Agroinfection of rice leaf tissues with** ***A. tumefaciens*** **suspension.** Histochemical assays to assess the expression of the *uidA* gene in rice tissues were carried out by staining with 5-bromo-4-chloro-3-indolyl-beta-D-glucuronid acid as described by Jefferson (1987). (**A**) Schematic representation of the small apparatus carrying many needles used to wound rice leaves. (**B**) Wounded rice leaves soaking in the *Agrobacterium* suspension in containment greenhouse. (**C**) GUS staining observed after 1, 3, 6 and 10 days post agroinfection (d.p.a) for three different cultivars of rice. No GUS staining was observed in unwounded and soaked leaves and in wounded but unsoaked leaves (not shown).

To establish an efficient method for *Agrobacterium* infection of rice leaves, we tested different *A. tumefaciens* strains (GV2200, EHA105, AGL1, LBA4404, and GV3101) carrying the same binary plasmid as well as different infiltration media and temperature, and assessed the efficiency of T-DNA transfer by expression of the carried *uidA* gene by GUS histochemical assays. The infiltration media and method of *Agrobacterium* preparation used for *Arabidopsis thaliana* floral dip or *Nicotiana* leaf agroinfiltration were completely inefficient on rice leaves whatever temperature or bacterial cell density applied (data not shown). Only the method of bacteria preparation used for genetic transformation of rice embryogenic calli yielded good results, using hyper-virulent strains such as EHA105, AGL1 or LBA4404. *Agrobacterium* cells were plated on solid AB medium containing antibiotic and incubated at 28°C for 3 days. The bacteria were then collected with a flat spatula and diluted in liquid agroinfection medium to optimal bacterial cell density ranging between 0.5 and 0.8. Because *A. tumefaciens* was not able to penetrate in the leaf through epidermal cuticle or via sub-stomatal chamber, we built a small apparatus carrying many 600 μm diameter needles (Figure 2A) to quickly produce a large number of wounds on the rice leaf surface. To avoid tissue yellowing or wilting, rice leaves were incubated in the *Agrobacterium* suspension for 30 to 60 minutes (Figure 2B). The first experiments, carried out in the containment greenhouse at 27-28°C, conducted to very weak *uidA* transgene expression, upon GUS histochemical assay (data not shown). Consequently, the temperature was reduced to 20°C during incubation. Moreover, supplementation of the agro-infection medium with Silwet L-77, a powerful surfactant, proved necessary to reach a high level, transgene expression. A two-fold concentration of acetosyringone (200 μM) also increased GUS staining intensity. For all rice genotype tested, high levels of GUS activity were observed 2 to 4 days following soaking in *Agrobacterium* cell suspensions of EHA105, LBA4404 and AGL1 strains. GUS staining was not detected any more after 10 days (Figure 2C). No GUS staining was observed in unwounded and soaked leaves and in wounded but unsoaked leaves (not shown). This protocol was also used successfully for transient expression of the *uidA* gene in the roots of rice plants grown under hydroponics (data not shown).

### Analysis of RNAi in HpRNA T-DNA stable transformants

**Figure 3:**
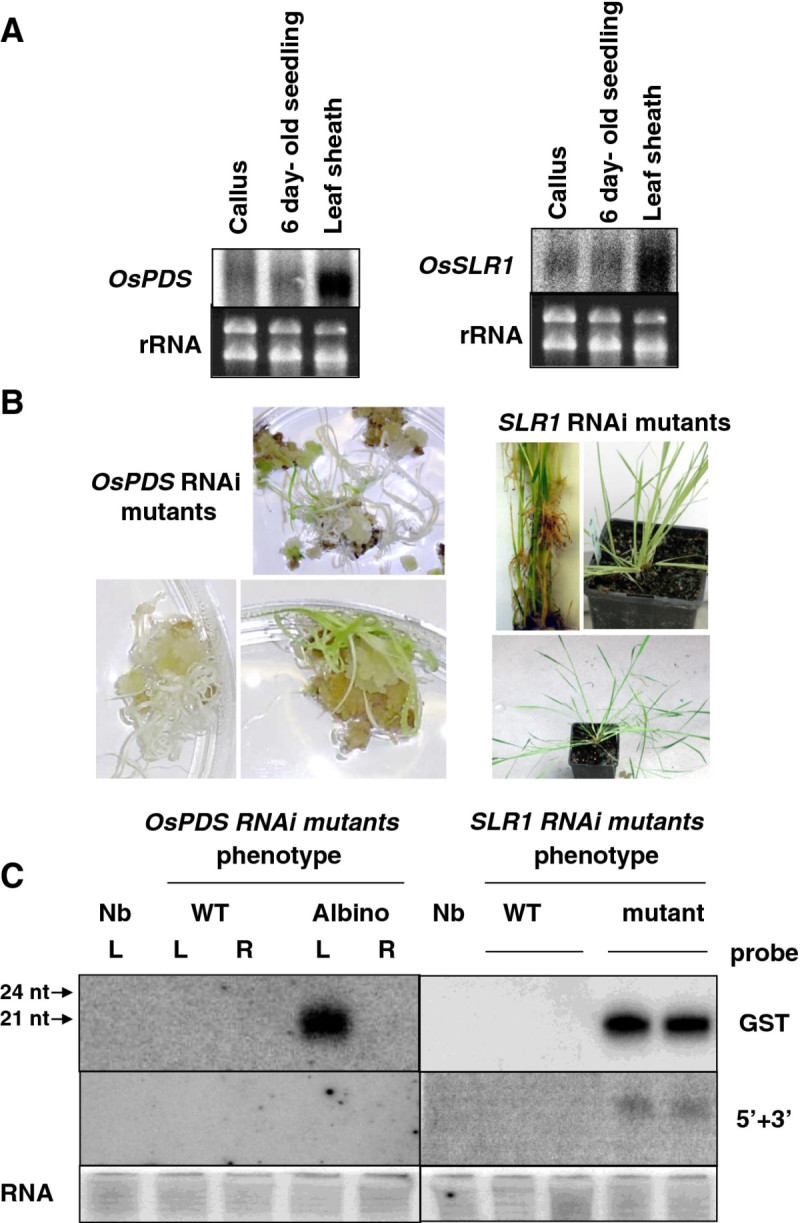
**Analysis of HpRNA T-DNA stable transformants.** (**A**) Northern blot detection of *OsPDS* and *OsSLR1* mRNA accumulation in different tissues of wild type plants of cv. Nipponbare. (**B**) Phenotypes of RNAi mutant lines expressing *OsPDS* RNAi HpRNA construct. (**C**) Detection of 21-nts siRNAs in *OsPDS* and *OsSLR1* HpRNA transgenic lines exhibiting or not a mutant phenotype. A probe specific to the gene specific tag (GST) and a probe representing regions extending 3’ and 5’ from the GST interval have been sequentially used. L (leaf), R (root), Nb (Control Nipponbare plant).

To determine whether RNA silencing can be initiated in agro-infected rice leaves by our method, we selected two endogenous gene targets namely Phytoene Desaturase (*OsPDS)* and *SLENDER 1* (*OsSLR1* or *OsGAI,* a rice ortholog of the height-regulating gene GAI/RGA/RHT/D8) because of their well-documented loss-of function phenotype. *PDS* has been used extensively to investigate gene silencing both in dicots (Voinnet 2001) and monocots (Miki et al. 2005). We first analyzed the endogenous mRNA level present in different tissues of wild type Nipponbare plants. We carried out northern blot analyses using the sequences of Os*PDS* and *OsSLR1* Gene Specific Tags (GST) as probes to hybridize sequentially the same membranes. In callus and leaves of six day-old seedlings, transcript accumulation of *OsPDS* and *OsSLR1* were rather similar. Contrastingly, in leaves of 5–6 leaf stage plantlets, *OsPDS* mRNAs accumulated at a higher level than those of *OsSLR1* (Figure 3A). To determine the efficacy of the hpRNA construct in triggering RNA silencing and thereby conducting to the anticipated loss of function phenotype, stable transgenic rice lines were produced through co-culture of 25 embryogenic calli of cv Nipponbare. Analysis of 85 *OsPDS* RNAi T0 lines plants showed various levels of *PDS* mRNA degradation, leading to phenotypes ranging from wild type to fully albino plants, in which the *PDS* gene was found to be strongly silenced (Figure 3B). Primary 21-nts-siRNAs, a molecular marker for dsRNA-based gene silencing, were easily detected in all albino lines which accounted for 54% of the regenerated plants (Figure 3C). A correlation was observed between the accumulation of siRNA and severity of the phenotype. Though a similar number of rice embryogenic calli was co-cultivated with *Agrobacterium* bearing the *OsSLR1* hpRNA construct, only four plants regenerated from transformed cell lines displayed a strong mutant phenotype but which sharply contrasted with that of *slr1* (Figure 3B) while other regenerated lines displayed a normal WT phenotype. Mutant plants were found to accumulate siRNAs whereas WT regenerants did not (Figure 3C**).** Based on the phenotype description of *slr1* mutants by Ikeda et al. (2001) we were indeed expecting a constitutive gibberellin response that leads to a tall phenotype. Though the *OsSLR1* is unique in the rice genome, the sequences of four other gene members of the GRAS family (namely *Os01g45860.1* also called *SLRL1*, *Os05g49990.1, Os11g03110.1* and *Os12g06540.1*) ( Additional file 1: Figure S1) share high similarity with a putative 21nt-siRNA residing in the Os*SLR1* GST sequence ( Additional file 2: Figure S2-A). This makes them candidates prone to targeting by *OsSLR1* primary siRNA that accumulate in the HpRNAi *slr1* mutants ( Additional file 2: Figure S2-B) through a mechanism called cross-silencing. Hybridization of small RNA blots of the HpRNAi *slr1* mutant with probes specific to each of these 4 genes evidenced accumulation of 21 nt siRNAs for 3 of them ( Additional file 2: Figure S2-C). It is therefore likely that this cross silencing accounts for the contrasting phenotype observed in our HpRNAi *slr1* mutant with regards to that of the reference disruption mutant.

### Analysis of the RNA silencing in leaves following agroinfection

**Figure 4:**
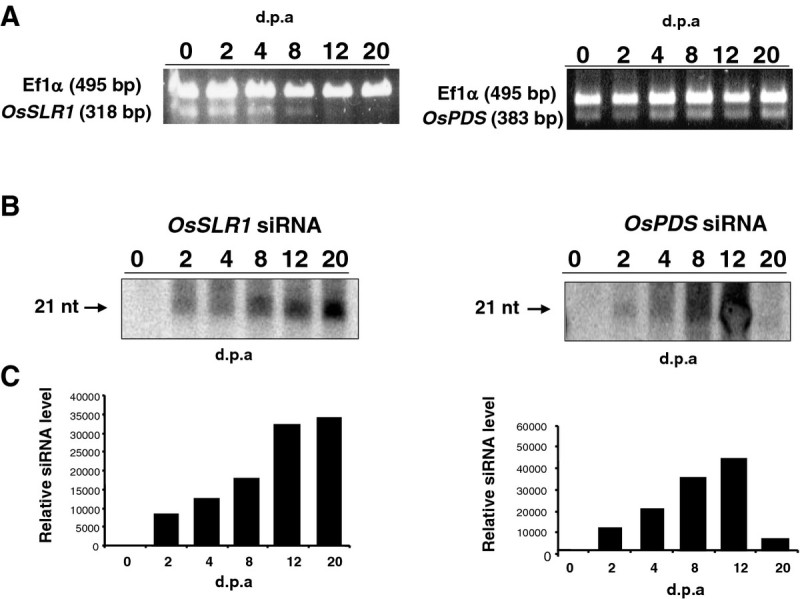
**Analysis of the agroinfiltrated leaf tissues.** (**A**) Accumulation of *OsPDS* and *OsSLR1* mRNAs revealed by RT-PCR during the time course experiment. *Ef1α* mRNA was used as a standard. (**B**) Time course of *OsPDS* and *OsSLR1* siRNAs accumulation following leaf agroinfection. Detection of siRNAs was performed using a GST probe. Arrow indicates the 21-nt-size-class siRNAs. Fifteen μg of total RNA extracted from leaf tissues after 2, 4, 8, 12 and 20 days were loaded on gel. A parallel time course was achieved with leaf tissues of plants agro-infiltrated with the pBIOS738 empty vector that served as reference value for siRNA accumulation. (**C**) Quantification of siRNAs detected by northern blotting using the ImageQuant™ TL image analysis software.

To determine whether agro-infection of leaf cells conducts to local induction of RNA silencing following transcription of the hpRNA construct, we agroinfected leaves of wild type Nipponbare plants using the T-DNA construct carrying the *OsPDS* and *OsSLR1* hpRNA structures. pBIOS738 empty vector was used as control over the time course experiment. RNA was isolated from the agro-infected regions of the leaf and analysed for the accumulation of transcripts and of siRNAs, 2, 4, 8, 12 and 20 days following inoculation (Figure 4A and B). Each time point involved at least three biological replicates. In the agro-infected region of the leaf, *OsPDS* and *OsSLR1* siRNAs were consistently detected from day 2 post-agroinfection. The image analysis software, ImageQuant™ TL (Amersham), was used to quantify siRNAs detected by northern blotting (Figure 4C). The *OsPDS* siRNAs concentration peaked at day 12 and dramatically decreased to reach a very low level at day 20, in the two first experiments. On the other hand, we were able to detect *OsPDS* siRNA 30 days after agro-infection in a third experiment (data not shown). Accumulation of the *OsSLR1* siRNAs was still occurring at day 20 though likely reaching a plateau. In each experiment, a population of plants agro-infected was further grown in the containment greenhouse and phenotypic observations were regularly performed. These plants displayed the same phenotype as the control plants throughout their life cycle. No siRNA could be detected in the tissues of non agro-infected leaves of higher and lower rank, whatever the hpRNA construct used, suggesting that very low or null long distance siRNA movement occurred through the phloem. In order to test whether the pattern of siRNA formation could be reproduced in indica rice, we repeated the agro-infection experiment with the same construct in leaves of cv. IR64. The siRNA accumulation pattern, analysed over 30 days post agro-infection, was fully consistent with that previously observed in the japonica cv. Nipponbare. Throughout the experiment and in both cultigens of rice, siRNA were not detected in the non agro-infected leaves.

### OsPDS and OsSRL1 silencing transitivity in rice leaf tissues

**Figure 5:**
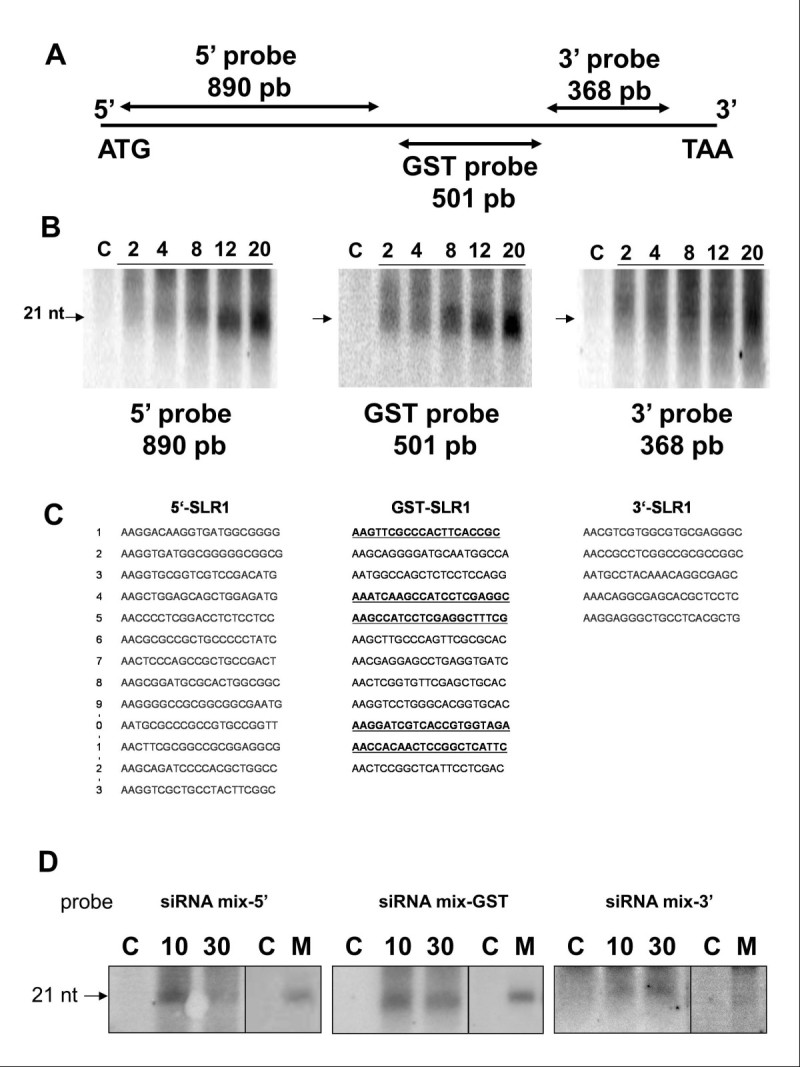
**Analysis of** ***OsSLR1*** **silencing transitivity in agro infiltrated leaf tissues.** (**A**) Schematic representation of the endogenous *OsSLR1* gene with size and position of the different probes used to detect primary and secondary siRNAs. (**B**) Time-course analysis of primary (GST probe) and secondary (5’ and 3’ probes) *OsSLR1* siRNA after rice leave agroinfection. (**C**) Sequences of potential siRNAs detected with Ambion software “siRNAs Target Finder” from 5’, GST and 3’ sequence part of *OsSLR1* coding sequence. (**D**) Time-course analysis of primary (putative siRNAs mix from GST as probe) and secondary (putative siRNAs mix from 5’ and 3’ part of *OsSLR1* coding sequence as probes) *OsSLR1* siRNA following rice leaves agroinfection and in *OsSLR1* RNAi mutant leaves (M).

In order to determine whether a transitive RNAi mechanism has been initiated in mutant lines and in agro-infected rice leaves, we hybridized the available blots with probes corresponding to regions the extending 5’ and 3’ from the trigger dsRNA interval in the two genes. In both agro-infected leaf areas and in stably transformed leaf tissues expressing the *OsPDS* hpRNA construct, siRNAs corresponding to the region used to produce the trigger dsRNA were detected but no siRNA was detected when the regions extending either 5’ or 3’ from the trigger dsRNA borders were used as probe (Figure 3C**)**. The membranes were re-hybridized with success using the trigger dsRNA probe to ascertain the presence of siRNAs on the filters following repeated cycles of hydridization-dehybridization. Contrastingly, siRNA corresponding to regions extending 5’ and 3’ from the trigger RNA interval accumulated in stably and transiently transformed leaf tissues expressing the *OsSLR1* hpRNA construct **(** Figures 3C and 5A and B). As the probes used were long the possibility of non-specific cross reaction between the 5’ and 3’ probes and the siRNAs derived from the silencing triggering hpRNA could not be ruled out. In order to ascertain hybridization specificity, the Ambion online siRNA target finder was used to identify all the potential siRNA from 5’, GST and 3’ part of *OsSLR1* coding sequence. Thirteen, 12, and 5 potential siRNAs were identified in these 3 respective regions **(** Figure 5C). It is assumed that nearly 50% of the siRNAs designed using this tool will achieve a >50% reduction of target gene expression. Considering this, oligo-nucleotides corresponding to each group were mixed in equimolar ratio and used as probe to re-hybridize small RNAs from leaves expressing the *OsSLR1* HpRNA construct (Figure 5D). Despite detection of lower signals, these experiments confirmed results obtained using longer probes. As a glimpse of Ambion software efficiency, potential siRNAs detected along the *OsSLR1* GST sequence were sequentially used as probe to re-hybridize RNAs extracted from *slr1* mutant lines. Five (41.6%) of 12 potential siRNAs showed a clear hybridization signal (data not shown).

### Variation of OsPDS transcript accumulation along the agroinfected leaf

**Figure 6:**
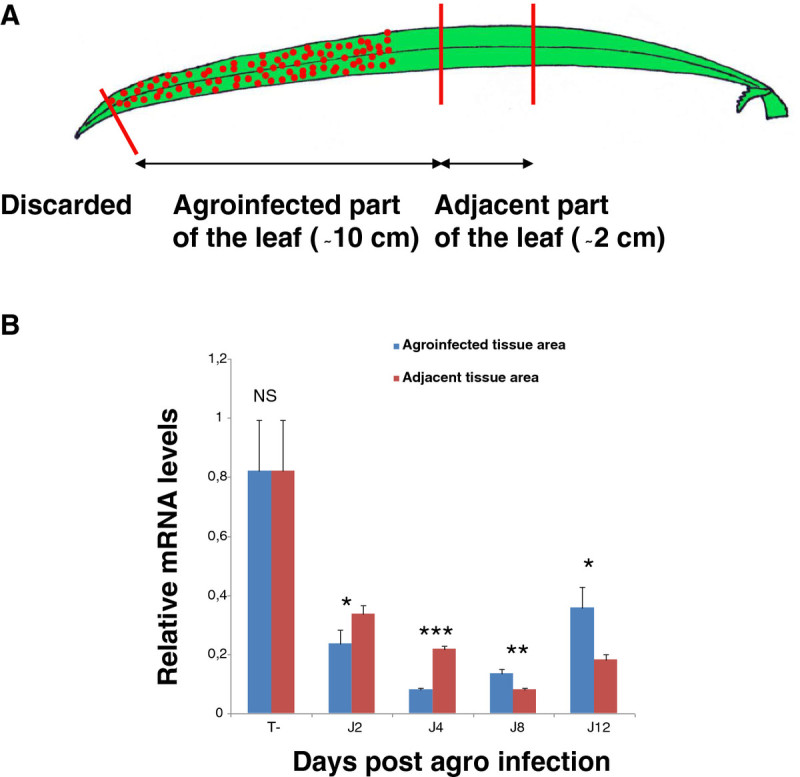
**Analysis of the movement of the** ***OsPDS*** **silencing signal from agroinfiltrated leaf tissues.** (**A**) Schematic representation of the rice leaf. Wounded and agroinfected part of the leaf and adjacent part are indicated. (**B**) Real-time PCR time course analysis of *OsPDS* transcript accumulation in the agro-infiltrated and adjacent region of the leaf. Results of 3 biological replicates are shown. Primer sequences were: *5’-pds*, 5’-GTTCCTGATCGAGTGAACGATG-3’, *3’-pds*, 5’-CGAACATGGTCAACAATAGGC-3’; *5’-OsExp*, 5’-CGGTTAGCTAGAGTTCATGTGAGA-3’, and *3’-OsExp*, 5’-ATTGGAGTAGTGGAGTGCCAAA-3’. NS, *, **,and *** means non significantly different and statistically different at the 5%, 1% and 0.1% level in a t-student test respectively.

To investigate the spreading of the RNAi silencing signal along the rice leaf, we compared the mRNA level of the target gene in agro-infected and adjacent leaf tissues by quantitative RT-PCR (Figure 6A). As the HpRNA construct-mediated silencing of the *OsSLR1* gene was prone to a complex cross silencing phenomenon that might be specific to the gene, we focused this experiment on tissues infiltrated with the *OsPDS* HpRNA construct. For q-RT-PCR, the gene of interest is co-amplified with an internal control gene to determine the relative abundance of target gene transcripts in the leaf tissue. *OsExp*, similar to its *Arabidopsis* ortholog (At4g33380), was chosen as the internal control because this gene showed good stability and its amplification remains in a log-linear stage at the same optimal conditions as those of target genes. Analysis of leaves agroinfected with the *OsPDS* hpRNA construct showed a close relationship between hpRNA transient expression and depression of PDS transcripts level. Compared to the wild type, the quantity of *OsPDS* mRNA fell to 29% two days after the agroinfection, and then reached a minimum of 10% at day 4. mRNA accumulation in agro-infected tissues reached 17% then 44% of the wild type value at day 8 and 12 respectively (Figure 6B). Similar results were observed for adjacent tissues with those agroinfected with a 4 day-shift for the peak of degradation of *OsPDS* mRNA which reaches 10% of the value of the wild type 8 days following agro-infection.

## Discussion

### An Agrobacterium-mediated transient expression system for cereal leaves

Because they provide a rapid, versatile and convenient way for achieving a very high level of gene expression in a distinct and defined zone of leaf, *Agrobacterium*-mediated transient expression systems have been chosen for inducing RNA silencing processes and transitivity analysis in leaves (Schöb et al. 1997). The first objective of this study was to evaluate the feasibility of an agro-infiltration approach for transient expression of a T-DNA construct in leaf tissues of rice. In cereal species, the leaf structure is known to prevent infiltration of a bacterial solution by simple pressure. In order to provide an access of *Agrobacterium* to the leaf cells, we used a combination of multiple uniform wound treatment and supplementation of the suspension with a powerful surfactant. After testing different *A. tumefaciens* strains as well as different infiltration media and temperatures, assessed by GUS histochemical assays, we have developed an efficient and reproducible method for agro-infection of leaves in indica and japonica rice, based on soaking wounded tissues in EHA105 bacterial solution at low temperature. The most critical physiological parameter appeared to be the post agro-infiltration incubation temperature. The largest and most intensively stained GUS areas in leaves were observed when rice plants are transferred at 20°C following agro-infiltration. A hypothesis is that a 25°C/28°C temperature favors bacterial multiplication *vs.* their attachment to plant cells and T-DNA transfer whereas at 20°C, bacterial multiplication slows down, thereby favoring plant cell-bacteria interactions. As reported in the literature in other plants (Batra and Kumar 2003; Wydro et al. 2006), we showed that acetosyringone was essential for efficient transfection, and the use of a two-fold concentration compared to our rice stable transformation procedure increases the efficiency of *in planta* agroinfection. As reported in other plants species (Wroblewski et al. 2005; Chabaud et al. 2003), the choice of the *Agrobacterium* strain appeared critical because of specific host-bacteria interactions (Tzfira and Citovsky 2002). In *Agrobacterium*–mediated *in planta* transformation, the genotype is generally found to influence T-DNA transfer and transgene expression. This was observed for instance in *Arabidopsis* and grapevine (Wroblewski et al. 2005; Zottini et al. 2008; Santos-Rosa et al. 2008). However, in our study, all the rice genotypes tested were found to be amenable to *Agrobacterium*-mediated transient expression of the *uidA* gene in infiltrated leaf cells. The optimized rice agroinfection protocol was also applied with success to sugarcane suggesting that other graminaceous species are amenable to this procedure (unpublished results). This method could be also very useful for transgenic complementation (Van der Hoorn et al. 2000; Shao et al. 2003; Bertazzon et al. 2012), promoter analysis (Yang et al. 2000), protein production (Vaquero et al. 1999) and ascertaining miRNA and amiRNA processing and target cleavage (Warthmann et al. 2008) in rice plants. The method is also applicable at different developmental stages as suggested by similar intensities of GUS staining observed in leaves of plants from the 5 leaf stage to the flowering stage. Our results indeed pointed that the developmental stage of the rice plants is not a critical parameter for an efficient expression of *uidA* gene induced in agroinfected leaves, though the efficiency becomes lower in old leaves than in seedling leaves probably due to plant resistance to agro-infection acquired at maturity (Wroblewski et al. 2005; Wydro et al. 2006; Santos-Rosa et al. 2008).

### HpRNA constructs triggers a maintained RNAi process in agro-infiltrated leaf tissues

In the experiments described here, we have analysed RNA silencing of two endogenous rice genes in both agro-infected leaf tissues and stably transformed rice plants, constitutively expressing an hpRNA construct. In both situations, we were able to detect siRNAs corresponding to the trigger dsRNA. In stable transgenic lines, the accumulation of which correlated the penetrance of the RNAi mutant phenotype, demonstrating the efficiency of our hpRNA construct in initiating RNA silencing of the corresponding endogenous gene in rice cells. Transient expression of hpRNA after leaf agro-infection was sufficient to trigger RNA silencing. During 10 to 20 days following rice agro-infection depending on the gene targeted, the quantity of siRNAs increased in cells then remains stable or decreased till leaf senescence. Considering that transient expression of the T-DNA-borne genes occurs only during the very few days following agroinfection (as attested by the revelation by histochemical assay of the otherwise rather stable GUS protein, that dramatically decreased between 6 to 10 days after agroinfection (data not shown)), we concluded that RNA silencing was maintained in rice cells in the absence of the trigger hpRNA. Local initiation of silencing produced preferentially primary 21-nt siRNA and maintenance of siRNAs production during cell life indicated that target genes are actively transcribed. This result is consistent with previously published data. 21-nt siRNAs are believed to guide mRNA cleavage, whereas 24-nt siRNAs are believed to exclusively mediate chromatin modification and transcriptional silencing by acting in a RISC-like complex (Brodersen and Voinnet 2006).

Real-time PCR analysis showed the highest *OsPDS* transcript depletion in the agro-infected leaf area 4 days following agro-infiltration. These results are consistent with the literature which reports a transitory peak of transgene expression at day 4 following agro-infection in *N. Benthamiana* and *A. thaliana.* Decrease of *OsPDS* mRNA degradation parallels the decline of transient expression of the HpRNA construct. Longer term maintenance of the silencing of the *OsPDS* gene and exhibition of the photobleaching phenotype would have probably required higher siRNA accumulation in agro infiltrated tissues. High level siRNA could be obtained in enhancing *Agrobacterium*-mediated delivery in leaf cells or through an amplification of the production of siRNA resulting from transitivity mechanism (see following section). Using the same hpRNA construct bearing the *OsPDS* GST sequence to initiate RNAi in tobacco leaves following agro-infection, we observed a similar amplification of the siRNAs and their maintenance in tissues where the initiation took place, but resulting in a photobleaching phenotype (unpublished results). The most obvious difference between rice and tobacco, at the experimental level, resides in the higher penetration of agrobacteria in leaves and consequently of bacterial cells in contact with leaf cells. In order to obtain an amplification of the degradation of the mRNA and to observe a phenotype in rice leaves, it might be necessary to increase the level of agro-infection in rice tissues.

### HpRNA-mediated induction of OsPDS and OsSLR1 silencing differs by the occurrence of transitivity in both stably and transiently transformed leaf tissues

We were not able to evidence transitivity in both transiently and stably silenced leaf tissues expressing the *OsPDS* HpRNA construct. This result is consistent with previous reports in tobacco (*rubisco* and *pds* genes; Vaistij et al. 2002), *Arabidopsis* (*rubisco* and *sulfur* genes; Himber et al. 2003) and rice (*OsGen-L***,** Moritoh et al. 2005; *Ospds* and *OsRAC* genes, Miki et al. 2005). Surprisingly, HpRNA-mediated silencing of the *OsSLR1* gene clearly involved transitivity with formation of siRNA occurring from regions of the endogenous target mRNA extending 5’ and 3’ from the region containing the trigger siRNA. One possible explanation for apparent lack of transitive RNA silencing of *OsPDS* endogenous gene may lie in a low concentration of the RNA substrates for RDR (Garcia-Perez et al. 2004). However, northern blot analysis of total RNA extracted from wild type plants clearly showed that transcription level of *OsPDS* is rather equivalent to that of *OsSLR1* in seedling leaves and is even higher at a later developmental stage. Along the same line, silencing of the most abundant mRNA in plants, that of the *rubisco* small subunit, does not involves transitivity nor long range spreading of the silencing signal (Vaistij et al. 2002; Himber et al. 2003) suggesting that the mRNA level might not be an essential factor in transitive RNA silencing. Experience on the RNA silencing transitivity in monocotyledonous plants is limited to the pioneering report of Miki et al. (2005) whose showed that the transitive RNA silencing machinery is conserved in rice. In this study, whereas transitive RNA silencing occurred in both 5’ and 3’ orientations for the exogenous *gfp* gene, no transitivity was observed for the endogenous *OsPDS* and *OsRAC* genes. Therefore, the cause of transitivity of the RNAi signal in *OsSLR1* remains to be further investigated.

### Spreading of the silencing signal to the non-agro infiltrated leaf tissues

Though the current model of cell-to-cell movement of the RNA silencing signal implies a central role for transitivity (Himber et al. 2003), we surprisingly detected an efficient decrease of the *OsPDS* mRNA level in the leaf tissue adjacent to the agroinfiltrated area occuring with a time lag. A possible explanation lies in the transfer of the siRNA from agro-infected tissues to healthy tissues through the symplastic pathway as described by Dunoyer et al. (2010). The vast majority of experimental data used to formulate the current model of cell-to-cell movement of RNA silencing signal was accumulated in *N. benthamiana* and *A. thaliana*. The short range movement (up to 10–15 cells) of the silencing signal requires accumulation of 21nt but not of 24nt siRNA while the possibility that ssRNA move from one cell to the other cannot be yet ruled out. The longer range (beyond 10–15 cells) systemic signal appears to be RDR6 dependent since no spreading and no secondary siRNA is observed in *rdr6* background. However, that long range spreading has so far been only observed in the RNAi of exogenous transgenes that generally implies extensive production of secondary siRNA and may result in exogenous DNA methylation. SiRNA cell-to-cell movements occur either in a non selective or selective manner through plasmodesmata (Hyun et al. 2011). It is thought that a molecular link exists between RNAi-mediated DNA methylation and regulation of selective siRNA movement through plasmodesmata but that link remains to be elucidated.

### Cross silencing occurred in HpRNA silencing of OsSLR1

As demonstrated by Miki et al. (2005) with the *OsRAC* gene family, a single IR of a specific region shared by several genes of a same family can simultaneously suppress the expression of multiple genes. In this work we studied the possibility of cross extinction of rice genes sharing sequence homology outside the sequence of *OsSLR1* GST. The results showed that transitivity could be responsible of multiple endogenous genes knock-down. So the choice of a highly conserved and specific sequence, is necessary to precisely suppress only one member in a gene family.

## Conclusion

In conclusion, this work demonstrates that *in planta*, *Agrobacterium*-mediated transient expression of T-DNA constructs is achievable in rice leaves. The possibility to trigger RNAi in a localized manner in the rice leaf opens new perspectives to study the cell-to-cell movement of the silencing signal in a monocot plant. This method could also be of particular interest to test the functionality of natural and artificial miRNA expression construct, in ascertaining miRNA processing and cleavage of target genes that are expressed in rice leaves. It is indeed often necessary to assay several amiRNAs, the sequences of which are based on computational predictions, to find one which effectively triggers knock down of a target gene (Warthmann et al. 2008). As this transfection method has been used to achieve localized GUS expression in roots of rice plants grown in hydroponics (data not shown) it should be as well applicable for target genes expressed in other rice organs.

Aurélie Andrieu and Jean Christophe Breitler are first co authors.

## Electronic supplementary material

Additional file 1:**Figure S1.** Phylogenetic tree of the members of the Arabidopsis thaliana and Oryza sativa GRAS transcription factor families generated through the GreenphylDB GOST tool (http://greenphyl.cirad.fr/v2/cgi-bin/index.cgi). Position of the four GRAS genes exhibiting a shared putative siRNA with *OsSLR1* are highlighted. (PPT 200 KB)

Additional file 2:**Figure S2. (A)** Alignments of the shared putative 21nt-siRNA residing in the sequence of OsSLR1and in those of the four other members of the GRAS family. **(B)** Detection of the common putative 21nt-siRNA in transformation events harbouring the *OsSLR1* HpRNA T-DNA construct and exhibiting (mutant) or not (wt) a phenotype. Nb: a control Nipponbare plant. **(C)** Detection of siRNAs specific to four other members of the GRAS family the sequence of which contains the putative, shared 21nt-siRNA in transformation events harbouring the *OsSLR1* HpRNA T-DNA construct and exhibiting (mutant) or not (wt) a phenotype. Nb: Control Nipponbare plant. (PPT 127 KB)

Below are the links to the authors’ original submitted files for images.

Authors’ original file for figure 1

Authors’ original file for figure 2

Authors’ original file for figure 3

Authors’ original file for figure 4

Authors’ original file for figure 5

Authors’ original file for figure 6

## References

[CR1] Batra S, Kumar S (2003). Agrobacterium-mediated transient GUS gene expression in buffel grass (Cenchrus ciliaris L.). J Appl Genet.

[CR2] Bertazzon N, Raiola A, Castiglioni C, Gardiman M, Angelini E, Borgo M, Ferrari S (2012). Transient silencing of the grapevine gene VvPGIP1 by agroinfiltration with a construct for RNA interference. Plant Cell Rep.

[CR3] Breitler JC, Meynard D, Van Boxtel J, Royer M, Bonnot F, Cambillau L, Guiderdoni E (2004). A novel 2 T-DNA binary vector allows efficient generation of marker-free transgenic plants in three elite cultivars of rice (Oryza sativa L.). Transgenic Res.

[CR4] Brodersen P, Voinnet O (2006). The diversity of RNA silencing pathways in plants. Trends Genet.

[CR5] Caldana C, Scheible W-R, Mueller-Roeber B, Ruzicic S (2007). A quantitative RT-PCR platform for high-throughput expression profiling of 2,500 rice transcription factors. Plant Methods.

[CR6] Chabaud M, De Carvalho-Niebel F, Barker DG (2003). Efficient transformation of Medicago truncatula cv. Jemlong using the hypervirulent Agrobacterium tumefaciens strain AGL1. Plant Cell Rep.

[CR7] Chilton M, Currier T, Farrand S, Bendich A, Gordon M, Nester E (1974). Agrobacterium tumefaciens DNA and PS8 bacteriophage DNA not detected in crown gall tumors. Proc Nat Acad Sci USA.

[CR8] Christensen A, Quail P (1996). Ubiquitin promoter-based vectors for high level expression of selectable and/or screenable marker genes in monocotyledonous plants. Transgenic Res.

[CR9] Dunoyer P, Schott G, Himber C, Meyer D, Takeda A, Carrington JC, Voinnet O (2010). Small RNA duplexes function as mobile silencing signals between plant cells. Science.

[CR10] Garcia-Perez R, Van Houdt H, Depicker A (2004). Spreading of post-transcriptional gene silencing along the target gene promotes systemic silencing. Plant J.

[CR11] Ghildiyal M, Zamore PD (2009). Small silencing RNAs an expanding universe. Nat Rev Genet.

[CR12] Himber C, Dunoyer P, Moissiard G, Ritzenthaler C, Voinnet O (2003). Transitivity-dependent and -independent cell-to-cell movement of RNA silencing. EMBO J.

[CR13] Hyun KT, Uddin MN, Rim Y, Kim JY (2011). Cell-to-cell trafficking of RNA and RNA silencing through plasmodesmata. Protoplasma.

[CR14] Ikeda A, Ueguchi-Tanaka M, Sonoda Y, Kitano H, Koshioka M, Futsuhara Y, Matsuoka M, Yamaguchi J (2001). Slender rice, a constitutive gibberellin response mutant, is caused by a null mutation of the SLR1 gene, an ortholog of the height-regulating gene GAI/RGA/RHT/D8. Plant Cell.

[CR15] Jefferson R (1987). Assaying chimeric genes in plants: The GUS gene fusion system. Plant Mol Biol Rep.

[CR16] Miki D, Itoh R, Shimamoto K (2005). RNA silencing of single and multiple members in a gene family of rice. Plant Physiol.

[CR17] Moritoh S, Miki D, Akiyama M, Kawahara M, Izawa T, Maki H, Shimamoto K (2005). RNAi-mediated silencing of OsGEN-L (OsGEN-like), a new member of the RAD2/XPG nuclease family, causes male sterility by defect of microspore development in rice. Plant Cell Physiol.

[CR18] Nishikura K (2001). A short primer on RNAi: RNA-directed RNA polymerase acts as a key catalyst. Cell.

[CR19] Sallaud C, Meynard D, van Boxtel J, Gay C, Bes M, Brizard JP, Larmande P, Ortega D, Raynal M, Portefaix M, Ouwerkerk PB, Rueb S, Delseny M, Guiderdoni E (2003). Highly efficient production and characterization of T-DNA plants for rice (Oryza sativa L.) functional genomics. Theor Appl Genet.

[CR20] Santos-Rosa M, Poutaraud A, Merdinoglu D, Mestre P (2008). Development of a transient expression system in grapevine via agro-infiltration. Plant Cell Rep.

[CR21] Shao F, Golstein C, Ade J, Stoutemyer M, Dixon JE, Innes RW (2003). Cleavage of Arabidopsis PBS1 by a bacterial type III effector. Science.

[CR22] Schöb H, Kunz C, Meins F (1997). Silencing of transgenes introduced into leaves by agroinfiltration: a simple, rapid method for investigating sequence requirements for gene silencing. Mol Gen Genet.

[CR23] Schweizer P, Pokorny J, Schulze-Lefert P, Dudler R (2000). Double-stranded RNA interferes with gene function at the single-cell level in cereals. Plant J.

[CR24] Sijen T, Fleenor J, Simmer F, Thijssen K, Parrish S, Timmons L, Plasterk R, Fire A (2001). On the role of RNA amplification in dsRNA-triggered gene silencing. Cell.

[CR25] Small I (2007). RNAi for revealing and engineering plant gene functions. Cur Opin Biotech.

[CR26] Tzfira T, Citovsky V (2002). Partners-in-infection: host proteins involved in the transformation of plant cells by Agrobacterium. Cell Biol.

[CR27] Vaistij FE, Jones L, Baulcombe DC (2002). Spreading of RNA targeting and DNA methylation in RNA silencing requires transcription of the target gene and a putative RNA-dependent RNA polymerase. Plant Cell.

[CR28] Vaquero C, Sack M, Chandler J, Drossard J, Schuster F, Monecke M, Schillberg S, Fischer R (1999). Transient expression of a tumor-specific single-chain fragment and a chimeric antibody in tobacco leaves. Proc Natl Acad Sci U S A.

[CR29] Van der Hoorn RA, Laurent F, Roth R, De Wit PJ (2000). Agroinfiltration is a versatile tool that facilitates comparative analyses of Avr9/Cf-9-induced and Avr4/Cf-4-induced necrosis. Mol Plant-Microbe Interact.

[CR30] Verdaguer B, de Kochko A, Fux CI, Beachy RN, Fauquet C (1998). Functional organization of the cassava vein mosaic virus (CsVMV) promoter. Plant Mol Biol.

[CR31] Voinnet O (2001). RNA silencing as a plant immune system against viruses. Trends in Genetics.

[CR32] Voinnet O (2005). Non-cell autonomous RNA silencing. FEBS Letters.

[CR33] Voinnet O (2005). Induction and suppression of RNA silencing: insights from viral infections. Nat Rev Genet.

[CR34] Warthmann N, Chen H, Ossowski S, Weigel D, Hervé P (2008). Highly Specific Gene Silencing by Artificial miRNAs in Rice. PLoS One.

[CR35] Wroblewski T, Tomczak A, Michelmore R (2005). Optimization of Agrobacterium -mediated transient assays of gene expression in lettuce, tomato and Arabidopsis. Plant Biotech J.

[CR36] Wydro M, Kozubek E, Lehmann P (2006). Optimization of transient Agrobacterium-mediated gene expression system in leaves of Nicotiana benthamiana. Acta Biochimica Polonica.

[CR37] Yang Y, Li R, Qi M (2000). In vivo analysis of plant promoters and transcription factors by agroinfiltration of tobacco leaves. Plant J.

[CR38] Zottini M, Barizza E, Costa A, Formentin E, Ruberti C, Carini F, Lo Schiavo F (2008). Agroinfiltration of grapevine leaves for fast transient assay of gene expression and for long-term production of stable transformed cells. Plant cell rep.

