# Supplementary figures and images for: An in planta, Agrobacterium-mediated transient gene expression method for inducing gene silencing in rice (Oryza sativa L.) leaves
Source: Rice (N Y). 2012 Aug 31;5:23. doi: 10.1186/1939-8433-5-23 (PMC4883685; doi:10.1186/1939-8433-5-23)

## Slide 1
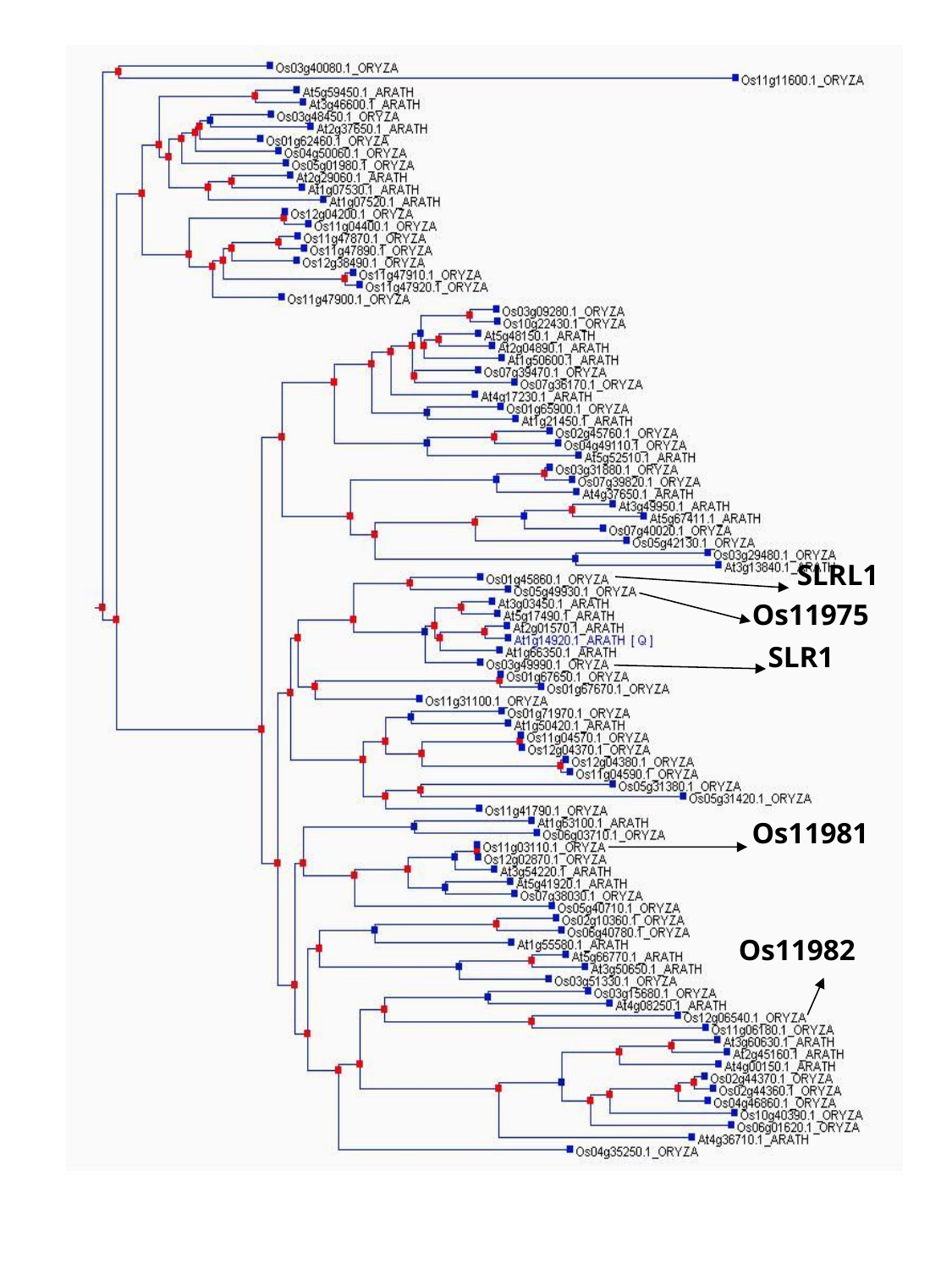

SLRL1
Os11975
SLR1
Os11981
Os11982
#

Supplement: Supplementary file 1 — Additional file 1:Figure S1. Phylogenetic tree of the members of the Arabidopsis thaliana and Oryza sativa GRAS transcription factor families generated through the GreenphylDB GOST tool (http://greenphyl.cirad.fr/v2/cgi-bin/index.cgi). Position of the four GRAS genes exhibiting a shared putative siRNA with OsSLR1 are highlighted. (PPT 200 KB) [file 12284_2011_26_MOESM1_ESM.ppt]

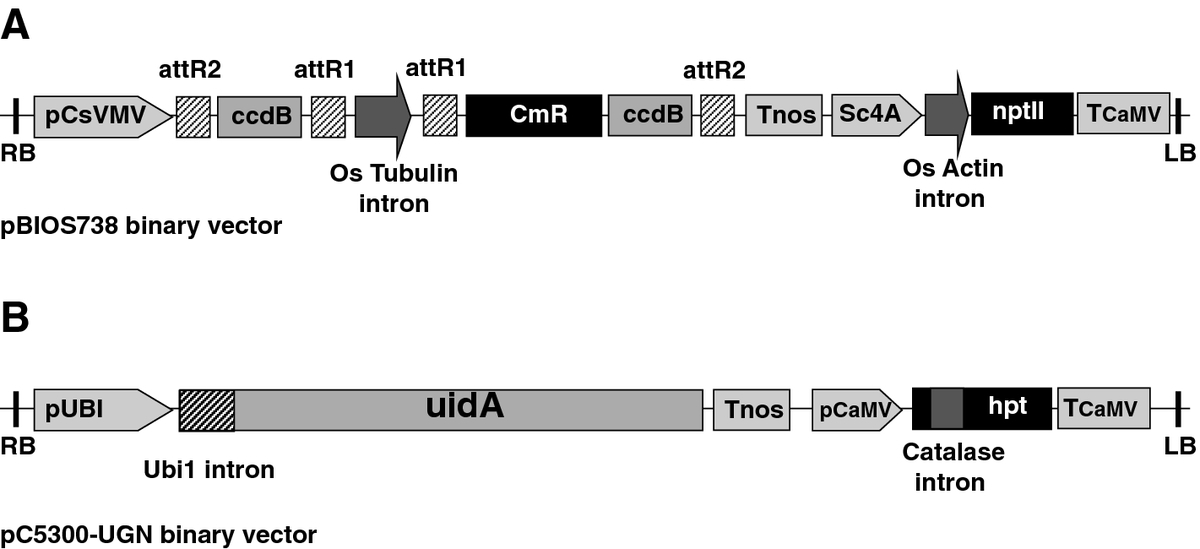

Supplement: Supplementary file 3 — Authors’ original file for figure 1 [file 12284_2011_26_MOESM3_ESM.jpeg]

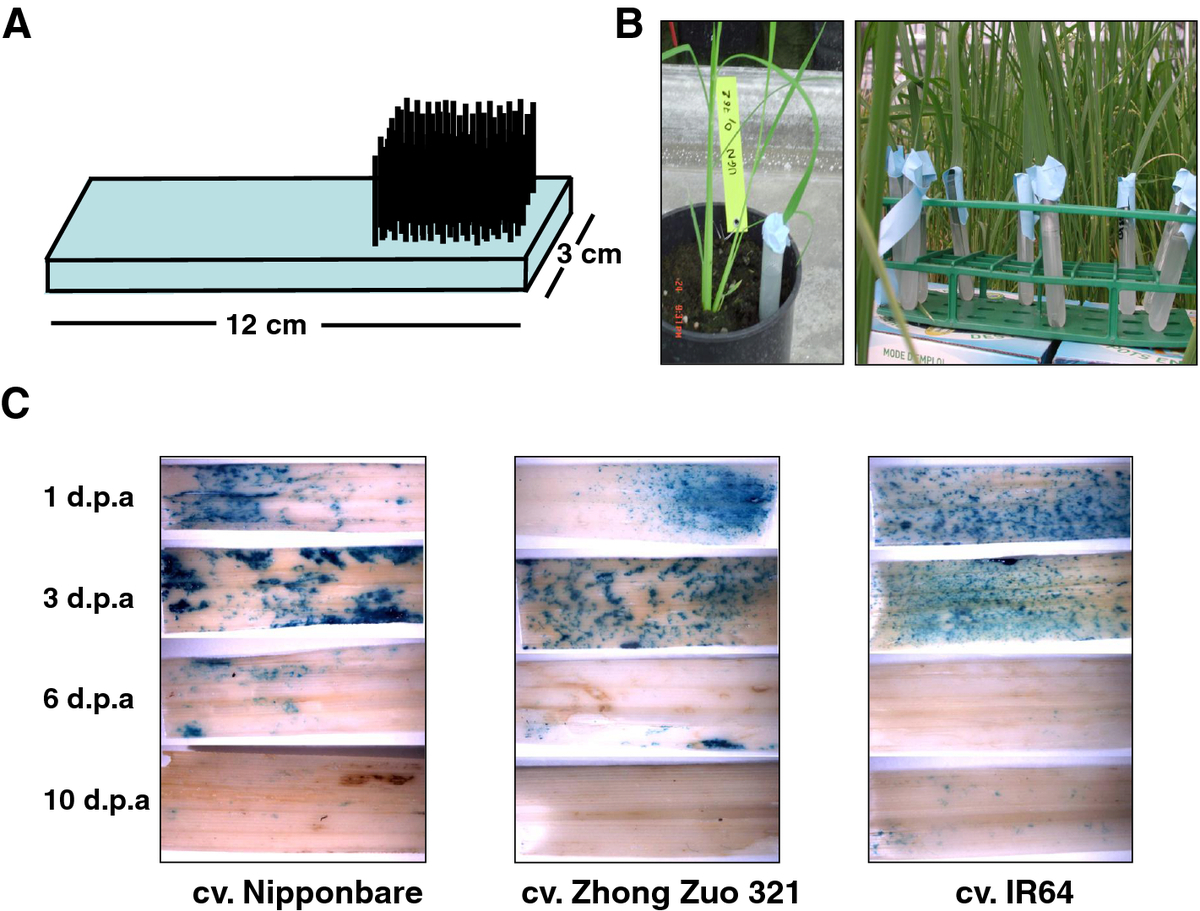

Supplement: Supplementary file 4 — Authors’ original file for figure 2 [file 12284_2011_26_MOESM4_ESM.jpeg]

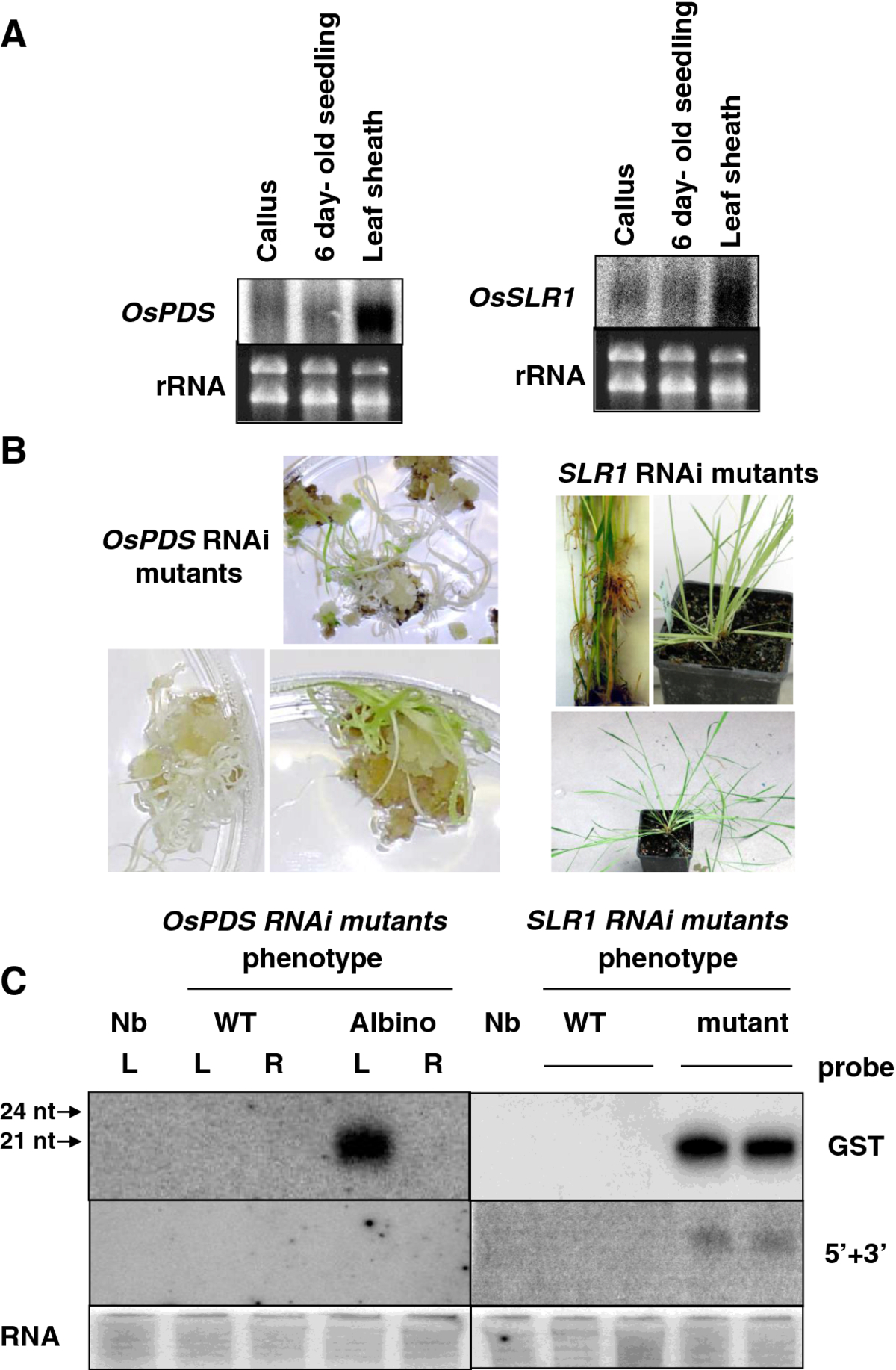

Supplement: Supplementary file 5 — Authors’ original file for figure 3 [file 12284_2011_26_MOESM5_ESM.jpeg]

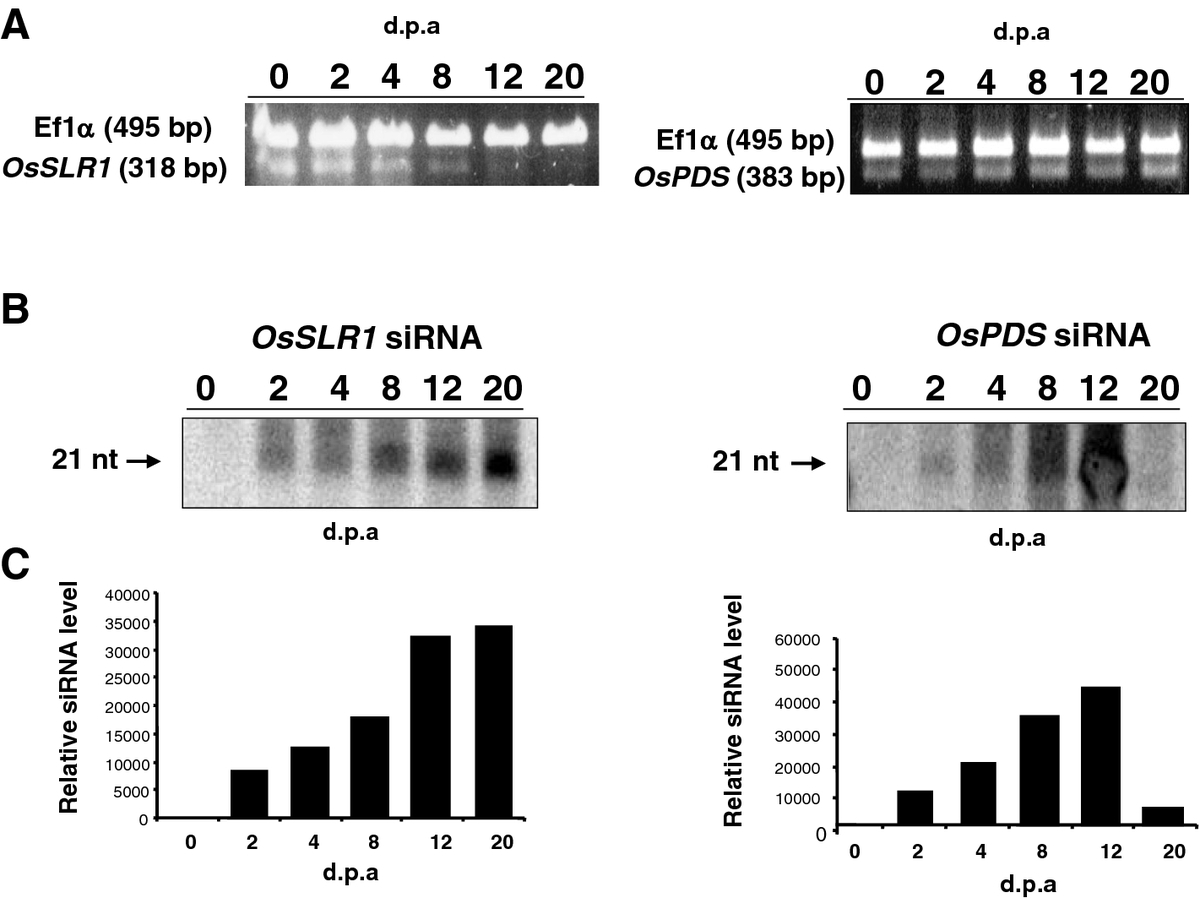

Supplement: Supplementary file 6 — Authors’ original file for figure 4 [file 12284_2011_26_MOESM6_ESM.jpeg]

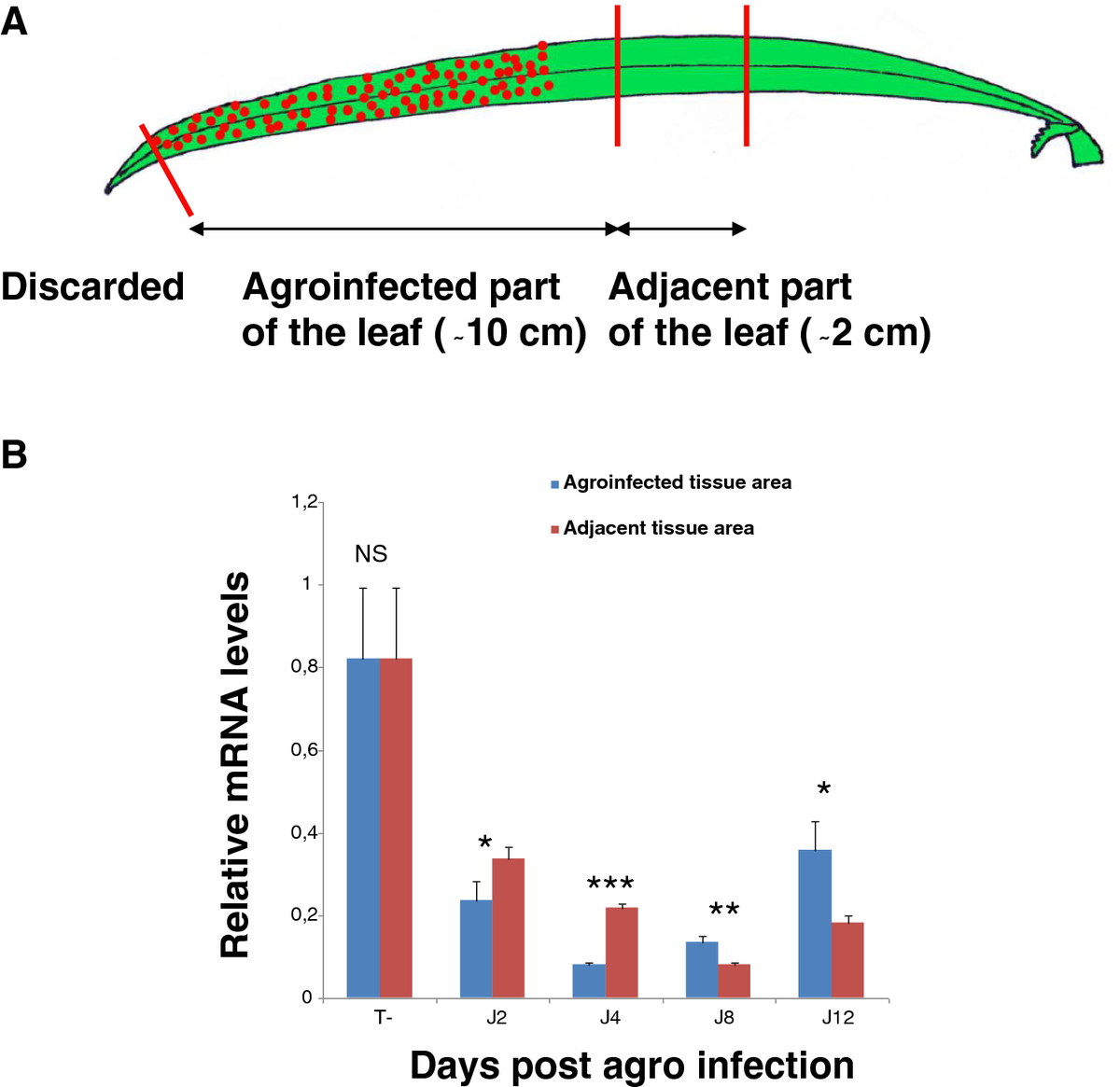

Supplement: Supplementary file 8 — Authors’ original file for figure 6 [file 12284_2011_26_MOESM8_ESM.jpeg]
