# Supplementary material for: An in planta, Agrobacterium-mediated transient gene expression method for inducing gene silencing in rice (Oryza sativa L.) leaves
Source: Rice (N Y). 2012 Aug 31;5:23. doi: 10.1186/1939-8433-5-23 (PMC4883685; doi:10.1186/1939-8433-5-23)
Supplement: Supplementary file 2 — Additional file 2:Figure S2. (A) Alignments of the shared putative 21nt-siRNA residing in the sequence of OsSLR1and in those of the four other members of the GRAS family. (B) Detection of the common putative 21nt-siRNA in transformation events harbouring the OsSLR1 HpRNA T-DNA construct and exhibiting (mutant) or not (wt) a phenotype. Nb: a control Nipponbare plant. (C) Detection of siRNAs specific to four other members of the GRAS family the sequence of which contains the putative, shared 21nt-siRNA in transformation events harbouring the OsSLR1 HpRNA T-DNA construct and exhibiting (mutant) or not (wt) a phenotype. Nb: Control Nipponbare plant. (PPT 127 KB) [file 12284_2011_26_MOESM2_ESM.ppt]

## Slide 1
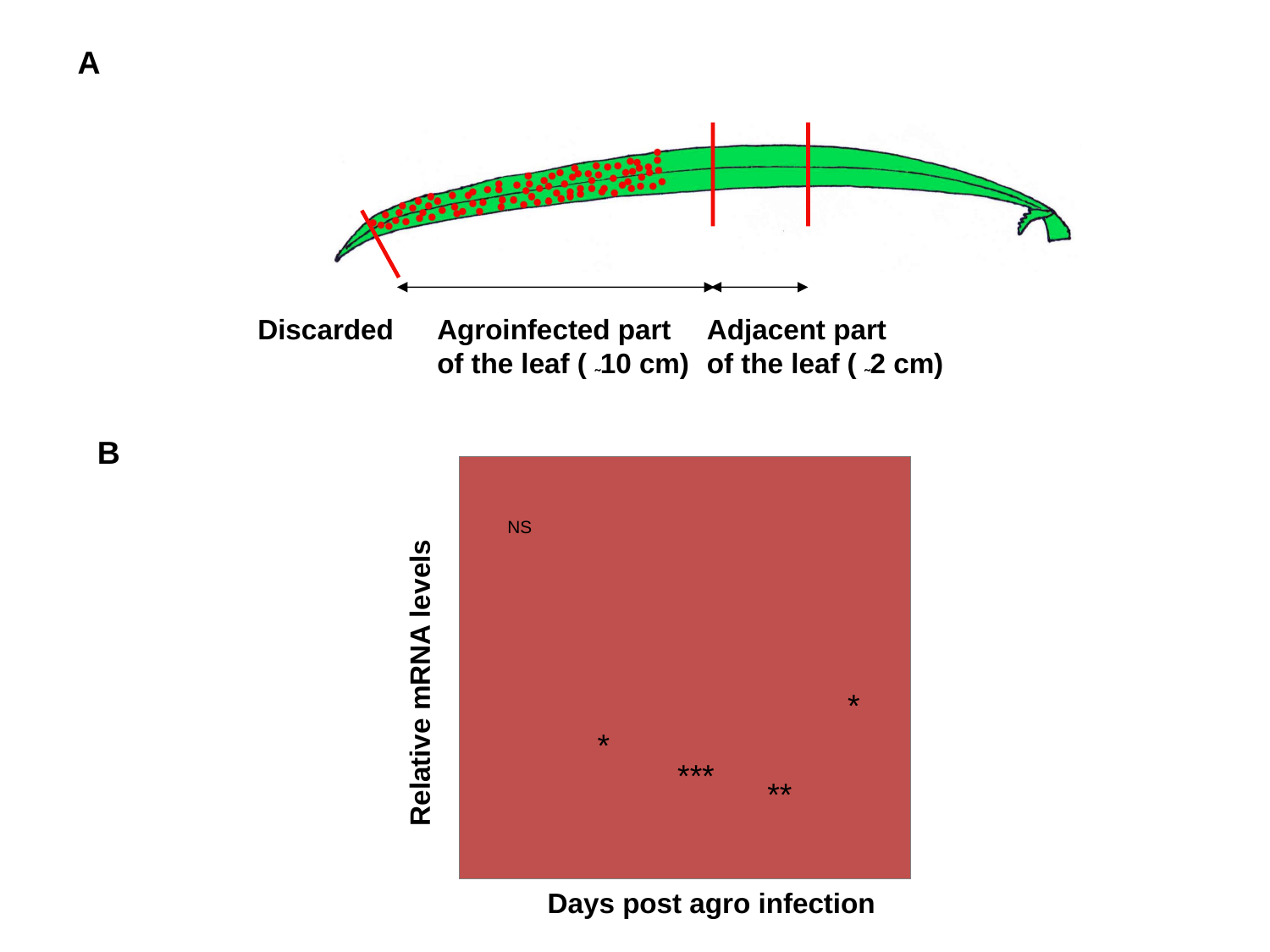

A
Discarded
Agroinfected part
of the leaf ( ˜10 cm)
Adjacent part
of the leaf ( ˜2 cm)
B
NS
Relative mRNA levels
*
*
***
**
Days post agro infection
