# Supplementary material for: An in planta, Agrobacterium-mediated transient gene expression method for inducing gene silencing in rice (Oryza sativa L.) leaves
Source: Rice (N Y). 2012 Aug 31;5:23. doi: 10.1186/1939-8433-5-23 (PMC4883685; doi:10.1186/1939-8433-5-23)
Supplement: Supplementary file 7 — Authors’ original file for figure 5 [file 12284_2011_26_MOESM7_ESM.ppt]

## Slide 1
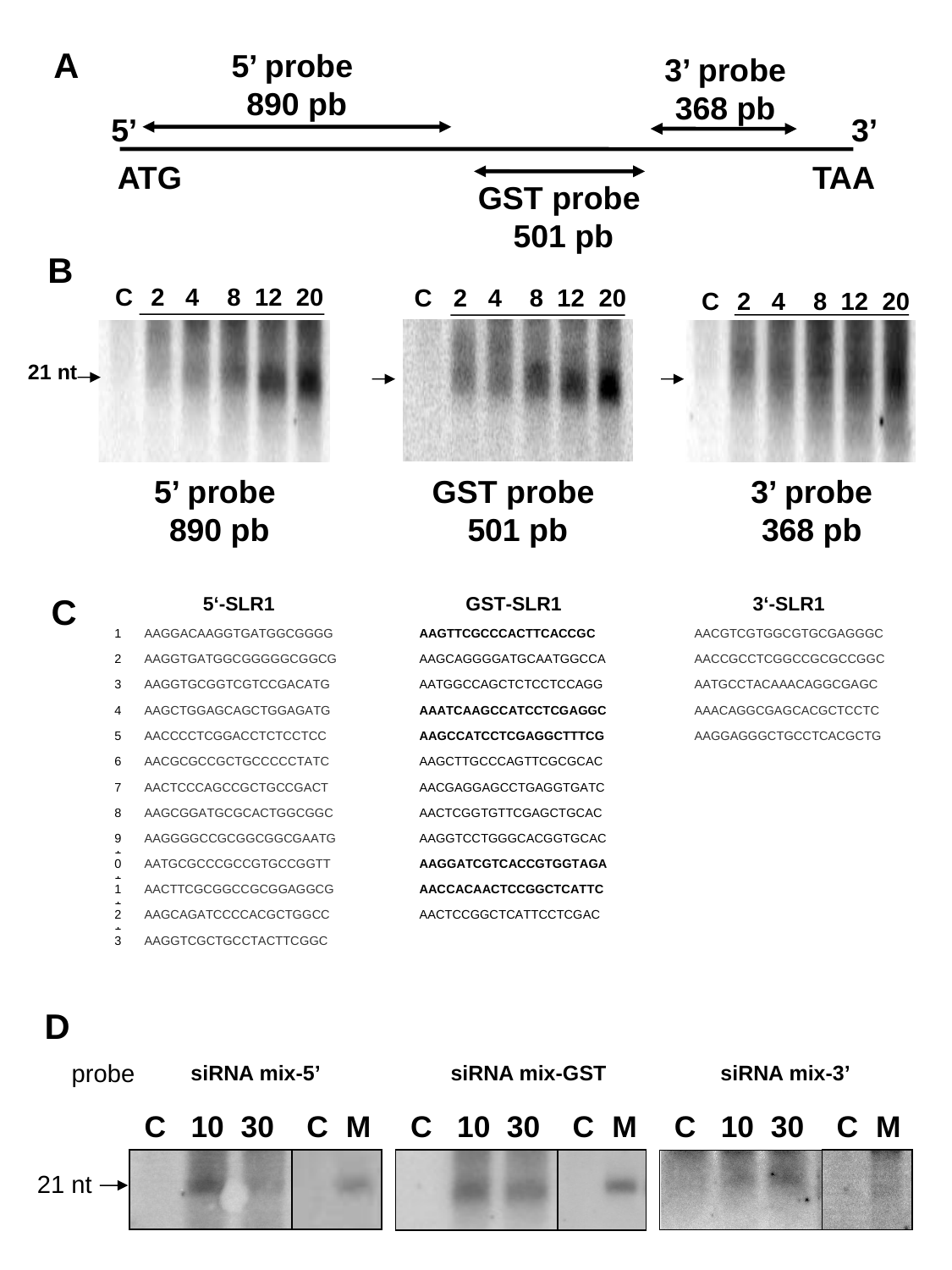

A
5’ probe
890 pb
3’ probe
368 pb
5’
3’
ATG
TAA
GST probe
501 pb
B
C
2 4 8 12 20
C
2 4 8 12 20
C
2 4 8 12 20
21 nt
5’ probe
890 pb
GST probe
501 pb
3’ probe
368 pb
C
D
probe
siRNA mix-5’
siRNA mix-GST
siRNA mix-3’
C
10 30
C
M
C
10 30
C
M
C
10 30
C
M
21 nt
